# Supplementary material for: The interactome of intact mitochondria by cross-linking mass spectrometry provides evidence for coexisting respiratory supercomplexes
Source: Mol Cell Proteomics. 2017 Dec 8;17(2):216–32. doi: 10.1074/mcp.RA117.000470 (PMC5795388; doi:10.1074/mcp.RA117.000470)
Supplement: Supplemental Data [file supp_RA117.000470_133922_0_supp_23978_fzffwf.zip › spectra_annotation/mito_spectra_annotation/444-1-7-1-10-1.pdf]

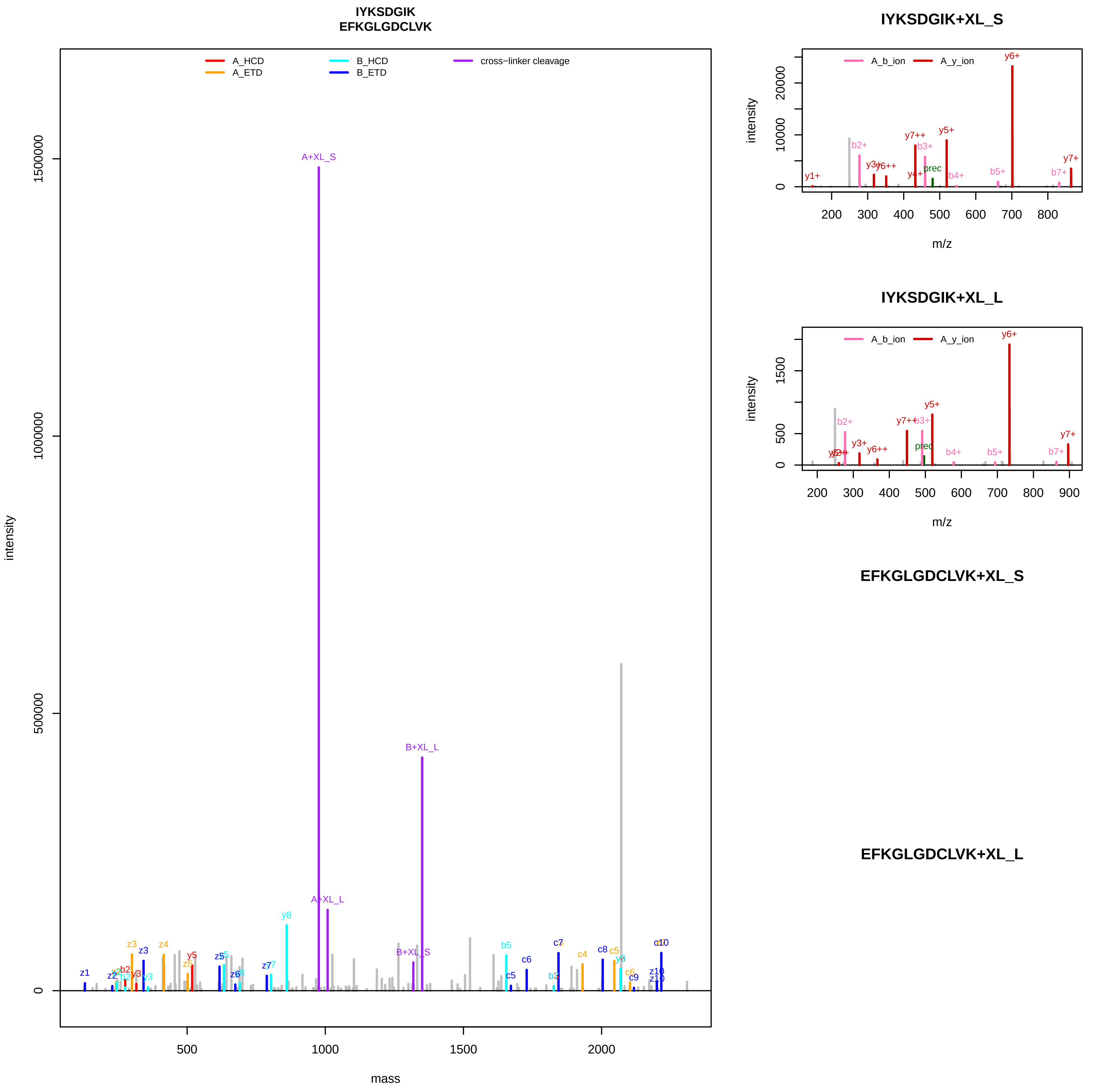

IYKSDGIK
EFKGLGDCLVK
A_HCD
A_ETD
B_HCD
B_ETD
cross−linker cleavage
A+XL_S
B+XL_L
A+XL_L
B+XL_S
intensity
mass
0
500000
1000000
1500000
500
1000
1500
2000
IYKSDGIK+XL_S
A_b_ion
A_y_ion
y6+
y7++
y5+
b2+
b3+
y3+
y6++
y4+
prec
b4+
b5+
b7+
y7+
y1+
intensity
m/z
0
10000
20000
200
300
400
500
600
700
800
IYKSDGIK+XL_L
A_b_ion
A_y_ion
intensity
m/z
0
500
1500
200
300
400
500
600
700
800
900
EFKGLGDCLVK+XL_S
EFKGLGDCLVK+XL_L
